# Dichlorido(2,9-dimethyl-1,10-phenanthroline-κ^2^ *N*,*N*′)cobalt(II)

**DOI:** 10.1107/S1600536810035531

**Published:** 2010-09-11

**Authors:** Niloufar Akbarzadeh Torbati, Ali Reza Rezvani, Nasser Safari, Vahid Amani, Hamid Reza Khavasi

**Affiliations:** a Department of Chemistry, University of Sistan and Baluchestan, PO Box 98135-674, Zahedan, Iran; b Department of Chemistry, Shahid Beheshti University, G. C., Evin, Tehran 1983963113, Iran

## Abstract

In the title compound, [CoCl_2_(C_14_H_12_N_2_)], the Co^II^ atom is four-coordinated in a distorted tetra­hedral geometry by two N atoms from a 2,9-dimethyl-1,10-phenanthroline ligand and two Cl atoms. The Co atom and the phenanthroline unit are located on a mirror plane. The methyl H atoms are disordered about the mirror plane and areeach half-occupied. In the crystal structure, π–π inter­actions between the pyridine and benzene rings and between the pyridine rings [centroid–centroid distances = 3.8821 (9) and 3.9502 (10) Å, respectively] stabilize the structure.

## Related literature

For related structures, see: Alizadeh *et al.* (2009 ▶); Buttery *et al.* (2006 ▶); Ding *et al.* (2006 ▶); Fanizzi *et al.* (1991 ▶); Lemoine *et al.* (2003 ▶); Preston & Kennard (1969 ▶); Robinson & Sinn (1975 ▶).
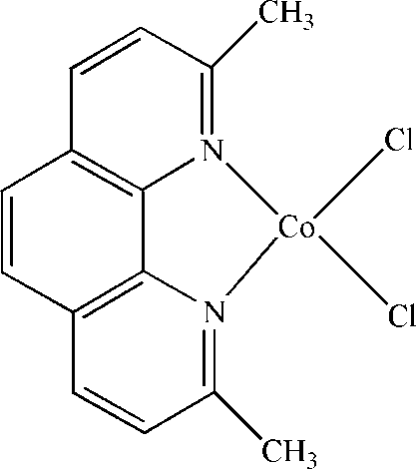


## Experimental

### 

#### Crystal data

[CoCl_2_(C_14_H_12_N_2_)]
*M* *_r_* = 338.09
Orthorhombic, 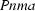

*a* = 11.2434 (12) Å
*b* = 7.441 (1) Å
*c* = 17.690 (3) Å
*V* = 1480.0 (4) Å^3^
*Z* = 4
Mo *K*α radiation
μ = 1.51 mm^−1^
*T* = 298 K
0.50 × 0.22 × 0.20 mm

#### Data collection

Stoe IPDS-2 diffractometer
Absorption correction: numerical (*X-SHAPE* and *X-RED*; Stoe & Cie, 2002 ▶) *T*_min_ = 0.681, *T*_max_ = 0.749
9742 measured reflections
2124 independent reflections
1871 reflections with *I* > 2σ(*I*)
*R*_int_ = 0.067

#### Refinement

*R*[*F* ^2^ > 2σ(*F* ^2^)] = 0.044
*wR*(*F* ^2^) = 0.139
*S* = 1.20
2124 reflections
114 parameters
H-atom parameters constrained
Δρ_max_ = 0.51 e Å^−3^
Δρ_min_ = −0.55 e Å^−3^

### 

Data collection: *X-AREA* (Stoe & Cie, 2002 ▶); cell refinement: *X-AREA*; data reduction: *X-RED* (Stoe & Cie, 2002 ▶); program(s) used to solve structure: *SHELXS97* (Sheldrick, 2008 ▶); program(s) used to refine structure: *SHELXL97* (Sheldrick, 2008 ▶); molecular graphics: *ORTEP-3* (Farrugia, 1997 ▶); software used to prepare material for publication: *WinGX* (Farrugia, 1999 ▶).

## Supplementary Material

Crystal structure: contains datablocks I, global. DOI: 10.1107/S1600536810035531/hy2350sup1.cif

Structure factors: contains datablocks I. DOI: 10.1107/S1600536810035531/hy2350Isup2.hkl

Additional supplementary materials:  crystallographic information; 3D view; checkCIF report

## Figures and Tables

**Table 1:** Selected bond lengths (Å)

N1—Co1	2.046 (3)
N2—Co1	2.046 (3)
Cl1—Co1	2.2030 (9)

**Fig. 1.**
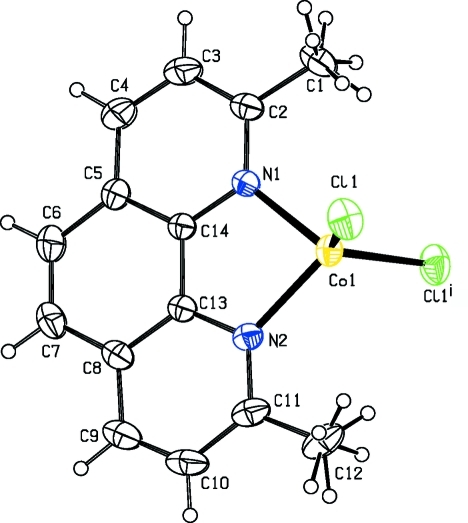
The molecular structure of the title compound. Displacement ellipsoids are drawn at the 30% probability level. [Symmetry code: (i) x, 3/2-y, z.]

**Fig. 2.**
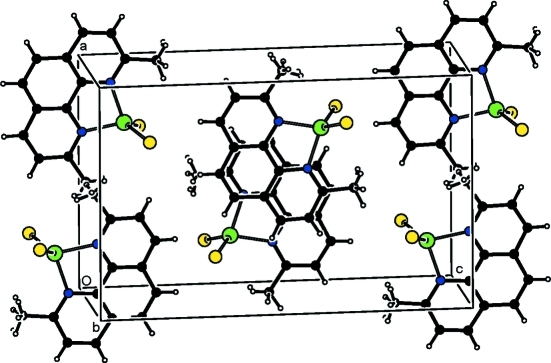
Crystal packing diagram for the title compound.

## supplementary crystallographic information

### Comment

2,9-Dimethyl-1,10-phenanthroline (dmphen) is a good bidentate ligand, and numerous complexes with dmphen have been prepared, such as those of mercury (Alizadeh *et al.*, 2009), zinc (Preston & Kennard, 1969), copper (Lemoine *et al.*, 2003), nickel (Ding *et al.*, 2006), gold (Robinson & Sinn, 1975), platinum (Fanizzi *et al.*, 1991) and lithium (Buttery *et al.*, 2006). Here, we report the synthesis and structure of the title compound.

In the title compound (Fig. 1), the Co^II^ atom is four-coordinated in a distorted tetrahedral configuration by two N atoms from one dmphen ligand and two Cl atoms (Table 1). In the crystal structure, π–π interactions (Fig. 2) between the pyridyl and benzene rings, *Cg*3···*Cg*4^i^ and *Cg*3···*Cg*3^ii^ [symmetry codes: (i) -*x*, 1-y, 2-*z*; (ii) -*x*, -1/2+y, 2-*z*; where *Cg*3 and *Cg*4 are the centroids of the N2, C8–C11, C13 ring and C5–C8, C13–C14 ring], with centroid–centroid distances of 3.8821 (9) and 3.9502 (10) Å, stabilize the structure.

### Experimental

For the preparation of the title compound, a solution of dmphen (0.42 g, 2.00 mmol) in methanol (20 ml) was added to a solution of CoCl_2_.6H_2_O (0.48 g, 2.00 mmol) in methanol (20 ml) at room temperature. Crystals suitable for X-ray diffraction analysis were obtained by methanol diffusion into a blue solution of the title compound in DMSO after one week (yield: 0.50 g, 73.9%; m.p. > 573 K).

### Refinement

H atoms were positioned geometrically and refined as riding atoms, with C—H = 0.93 (aromatic) and 0.96 (methyl) Å and with *U*_iso_(H) = 1.2*U*_eq_(C).

### Crystal data

**Table:**

[CoCl_2_(C_14_H_12_N_2_)]	*F*(000) = 684
*M**_r_* = 338.09	*D*_x_ = 1.517 Mg m^−^^3^
Orthorhombic, *P**n**m**a*	Mo *K*α radiation, λ = 0.71073 Å
Hall symbol: -P 2ac 2n	Cell parameters from 1670 reflections
*a* = 11.2434 (12) Å	θ = 2.2–29.3°
*b* = 7.441 (1) Å	µ = 1.51 mm^−^^1^
*c* = 17.690 (3) Å	*T* = 298 K
*V* = 1480.0 (4) Å^3^	Block, blue
*Z* = 4	0.50 × 0.22 × 0.20 mm

### Data collection

**Table:**

Stoe IPDS-2 diffractometer	2124 independent reflections
Radiation source: fine-focus sealed tube	1871 reflections with *I* > 2σ(*I*)
graphite	*R*_int_ = 0.067
rotation method scans	θ_max_ = 29.3°, θ_min_ = 2.2°
Absorption correction: numerical (*X-SHAPE* and *X-RED*; Stoe & Cie, 2002)	*h* = −14→15
*T*_min_ = 0.681, *T*_max_ = 0.749	*k* = −10→10
9742 measured reflections	*l* = −15→24

### Refinement

**Table:**

Refinement on *F*^2^	Primary atom site location: structure-invariant direct methods
Least-squares matrix: full	Secondary atom site location: difference Fourier map
*R*[*F*^2^ > 2σ(*F*^2^)] = 0.044	Hydrogen site location: inferred from neighbouring sites
*wR*(*F*^2^) = 0.139	H-atom parameters constrained
*S* = 1.20	*w* = 1/[σ^2^(*F*_o_^2^) + (0.0692*P*)^2^ + 0.4334*P*] where *P* = (*F*_o_^2^ + 2*F*_c_^2^)/3
2124 reflections	(Δ/σ)_max_ = 0.001
114 parameters	Δρ_max_ = 0.51 e Å^−^^3^
0 restraints	Δρ_min_ = −0.55 e Å^−^^3^

### Fractional atomic coordinates and isotropic or equivalent isotropic displacement parameters (Å^2^)

**Table:**

	*x*	*y*	*z*	*U*_iso_*/*U*_eq_	Occ. (<1)
C1	0.5096 (3)	0.7500	0.9952 (3)	0.0677 (12)	
H1A	0.4953	0.6880	0.9484	0.102*	0.50
H1B	0.5719	0.6904	1.0224	0.102*	0.50
H1C	0.5329	0.8716	0.9849	0.102*	0.50
C2	0.3980 (3)	0.7500	1.0417 (2)	0.0502 (8)	
C3	0.4022 (4)	0.7500	1.1208 (3)	0.0670 (12)	
H3	0.4754	0.7500	1.1454	0.080*	
C4	0.3002 (4)	0.7500	1.1619 (2)	0.0661 (11)	
H4	0.3038	0.7500	1.2145	0.079*	
C5	0.1893 (3)	0.7500	1.1254 (2)	0.0470 (7)	
C6	0.0776 (4)	0.7500	1.1635 (2)	0.0599 (10)	
H6	0.0763	0.7500	1.2161	0.072*	
C7	−0.0259 (4)	0.7500	1.1254 (3)	0.0602 (10)	
H7	−0.0974	0.7500	1.1518	0.072*	
C8	−0.0270 (3)	0.7500	1.0444 (2)	0.0486 (8)	
C9	−0.1318 (3)	0.7500	1.0008 (3)	0.0648 (11)	
H9	−0.2057	0.7500	1.0244	0.078*	
C10	−0.1246 (4)	0.7500	0.9245 (3)	0.0687 (12)	
H10	−0.1939	0.7500	0.8959	0.082*	
C11	−0.0128 (4)	0.7500	0.8877 (2)	0.0556 (9)	
C12	−0.0018 (5)	0.7500	0.8039 (3)	0.0775 (14)	
H12A	−0.0718	0.8026	0.7821	0.116*	0.50
H12B	0.0067	0.6287	0.7861	0.116*	0.50
H12C	0.0668	0.8187	0.7894	0.116*	0.50
C13	0.0806 (3)	0.7500	1.00504 (19)	0.0393 (6)	
C14	0.1911 (3)	0.7500	1.04563 (19)	0.0387 (6)	
N1	0.2938 (2)	0.7500	1.00521 (16)	0.0392 (5)	
N2	0.0873 (2)	0.7500	0.92773 (17)	0.0423 (6)	
Cl1	0.31373 (7)	0.49607 (9)	0.83732 (5)	0.0645 (2)	
Co1	0.26015 (4)	0.7500	0.89154 (3)	0.04234 (18)	

### Atomic displacement parameters (Å^2^)

**Table:**

	*U*^11^	*U*^22^	*U*^33^	*U*^12^	*U*^13^	*U*^23^
C1	0.0351 (16)	0.082 (3)	0.086 (3)	0.000	0.0071 (19)	0.000
C2	0.0353 (14)	0.058 (2)	0.058 (2)	0.000	−0.0052 (14)	0.000
C3	0.050 (2)	0.093 (3)	0.058 (2)	0.000	−0.0196 (18)	0.000
C4	0.062 (2)	0.093 (3)	0.043 (2)	0.000	−0.0126 (18)	0.000
C5	0.0494 (18)	0.0531 (18)	0.0387 (16)	0.000	0.0011 (13)	0.000
C6	0.064 (2)	0.073 (2)	0.0425 (19)	0.000	0.0116 (17)	0.000
C7	0.0507 (19)	0.070 (2)	0.060 (2)	0.000	0.0188 (17)	0.000
C8	0.0363 (15)	0.0488 (17)	0.061 (2)	0.000	0.0026 (14)	0.000
C9	0.0357 (16)	0.070 (3)	0.088 (3)	0.000	−0.0046 (18)	0.000
C10	0.0415 (18)	0.075 (3)	0.089 (3)	0.000	−0.024 (2)	0.000
C11	0.056 (2)	0.0505 (19)	0.061 (2)	0.000	−0.0229 (17)	0.000
C12	0.096 (4)	0.087 (3)	0.049 (2)	0.000	−0.033 (2)	0.000
C13	0.0353 (13)	0.0386 (13)	0.0441 (16)	0.000	−0.0016 (12)	0.000
C14	0.0353 (13)	0.0421 (14)	0.0388 (14)	0.000	−0.0013 (11)	0.000
N1	0.0331 (11)	0.0438 (13)	0.0409 (14)	0.000	−0.0001 (10)	0.000
N2	0.0413 (13)	0.0436 (13)	0.0420 (14)	0.000	−0.0063 (11)	0.000
Cl1	0.0738 (5)	0.0488 (4)	0.0707 (5)	0.0020 (3)	0.0176 (4)	−0.0121 (3)
Co1	0.0470 (3)	0.0423 (3)	0.0378 (3)	0.000	0.00572 (17)	0.000

### Geometric parameters (Å, °)

**Table:**

C1—C2	1.500 (5)	C8—C9	1.408 (5)
C1—H1A	0.9600	C9—C10	1.352 (8)
C1—H1B	0.9600	C9—H9	0.9300
C1—H1C	0.9600	C10—C11	1.416 (7)
C2—N1	1.337 (4)	C10—H10	0.9300
C2—C3	1.401 (6)	C11—N2	1.330 (4)
C3—C4	1.358 (7)	C11—C12	1.487 (6)
C3—H3	0.9300	C12—H12A	0.9600
C4—C5	1.405 (6)	C12—H12B	0.9600
C4—H4	0.9300	C12—H12C	0.9600
C5—C14	1.411 (5)	C13—N2	1.370 (4)
C5—C6	1.426 (5)	C13—C14	1.434 (4)
C6—C7	1.345 (6)	C14—N1	1.359 (4)
C6—H6	0.9300	N1—Co1	2.046 (3)
C7—C8	1.433 (6)	N2—Co1	2.046 (3)
C7—H7	0.9300	Cl1—Co1	2.2030 (9)
C8—C13	1.396 (5)	Co1—Cl1^i^	2.2030 (9)
			
C2—C1—H1A	109.5	C9—C10—C11	120.9 (4)
C2—C1—H1B	109.5	C9—C10—H10	119.6
H1A—C1—H1B	109.5	C11—C10—H10	119.6
C2—C1—H1C	109.5	N2—C11—C10	120.4 (4)
H1A—C1—H1C	109.5	N2—C11—C12	117.4 (4)
H1B—C1—H1C	109.5	C10—C11—C12	122.2 (4)
N1—C2—C3	120.8 (4)	C11—C12—H12A	109.5
N1—C2—C1	117.9 (4)	C11—C12—H12B	109.5
C3—C2—C1	121.4 (4)	H12A—C12—H12B	109.5
C4—C3—C2	120.4 (4)	C11—C12—H12C	109.5
C4—C3—H3	119.8	H12A—C12—H12C	109.5
C2—C3—H3	119.8	H12B—C12—H12C	109.5
C3—C4—C5	120.2 (4)	N2—C13—C8	123.0 (3)
C3—C4—H4	119.9	N2—C13—C14	116.9 (3)
C5—C4—H4	119.9	C8—C13—C14	120.1 (3)
C4—C5—C14	116.6 (3)	N1—C14—C5	122.6 (3)
C4—C5—C6	124.3 (4)	N1—C14—C13	118.2 (3)
C14—C5—C6	119.1 (3)	C5—C14—C13	119.2 (3)
C7—C6—C5	121.6 (4)	C2—N1—C14	119.4 (3)
C7—C6—H6	119.2	C2—N1—Co1	129.5 (2)
C5—C6—H6	119.2	C14—N1—Co1	111.1 (2)
C6—C7—C8	120.6 (3)	C11—N2—C13	119.1 (3)
C6—C7—H7	119.7	C11—N2—Co1	129.6 (3)
C8—C7—H7	119.7	C13—N2—Co1	111.3 (2)
C13—C8—C9	116.9 (4)	N1—Co1—N2	82.44 (11)
C13—C8—C7	119.4 (3)	N1—Co1—Cl1	112.17 (4)
C9—C8—C7	123.7 (4)	N2—Co1—Cl1	113.31 (4)
C10—C9—C8	119.7 (4)	N1—Co1—Cl1^i^	112.17 (4)
C10—C9—H9	120.1	N2—Co1—Cl1^i^	113.34 (4)
C8—C9—H9	120.1	Cl1—Co1—Cl1^i^	118.12 (5)
			
N1—C2—C3—C4	0.000 (3)	C1—C2—N1—C14	180.000 (1)
C1—C2—C3—C4	180.000 (2)	C3—C2—N1—Co1	180.000 (1)
C2—C3—C4—C5	0.000 (3)	C1—C2—N1—Co1	0.000 (2)
C3—C4—C5—C14	0.000 (2)	C5—C14—N1—C2	0.000 (2)
C3—C4—C5—C6	180.000 (2)	C13—C14—N1—C2	180.000 (1)
C4—C5—C6—C7	180.000 (2)	C5—C14—N1—Co1	180.000 (1)
C14—C5—C6—C7	0.000 (3)	C13—C14—N1—Co1	0.000 (1)
C5—C6—C7—C8	0.000 (3)	C10—C11—N2—C13	0.000 (2)
C6—C7—C8—C13	0.000 (2)	C12—C11—N2—C13	180.000 (2)
C6—C7—C8—C9	180.000 (2)	C10—C11—N2—Co1	180.000 (1)
C13—C8—C9—C10	0.000 (2)	C12—C11—N2—Co1	0.000 (1)
C7—C8—C9—C10	180.000 (2)	C8—C13—N2—C11	0.000 (2)
C8—C9—C10—C11	0.000 (2)	C14—C13—N2—C11	180.000 (1)
C9—C10—C11—N2	0.000 (2)	C8—C13—N2—Co1	180.000 (1)
C9—C10—C11—C12	180.000 (2)	C14—C13—N2—Co1	0.000 (1)
C9—C8—C13—N2	0.000 (2)	C2—N1—Co1—N2	180.000 (1)
C7—C8—C13—N2	180.000 (2)	C14—N1—Co1—N2	0.000 (1)
C9—C8—C13—C14	180.000 (1)	C2—N1—Co1—Cl1	67.86 (4)
C7—C8—C13—C14	0.000 (2)	C14—N1—Co1—Cl1	−112.14 (4)
C4—C5—C14—N1	0.000 (2)	C2—N1—Co1—Cl1^i^	−67.82 (4)
C6—C5—C14—N1	180.000 (2)	C14—N1—Co1—Cl1^i^	112.18 (4)
C4—C5—C14—C13	180.000 (2)	C11—N2—Co1—N1	180.000 (1)
C6—C5—C14—C13	0.000 (2)	C13—N2—Co1—N1	0.000 (1)
N2—C13—C14—N1	0.000 (2)	C11—N2—Co1—Cl1	−69.07 (5)
C8—C13—C14—N1	180.000 (1)	C13—N2—Co1—Cl1	110.93 (5)
N2—C13—C14—C5	180.000 (2)	C11—N2—Co1—Cl1^i^	69.07 (5)
C8—C13—C14—C5	0.000 (2)	C13—N2—Co1—Cl1^i^	−110.93 (5)
C3—C2—N1—C14	0.000 (2)		

Symmetry codes: (i) *x*, −*y*+3/2, *z*.
